# Railway Travellng and Abortion

**Published:** 1844-07-27

**Authors:** 


RAILWAY TRAVELLNG AND ABORTION.—Under this very extraordinary head, Mr. Rankin writes a letter to shew that in Scotland, at least, abortion has been more frequent since the introduction of railways, and he is desirous of ascertaining whether a similar result has been produced in England. The question is one of considerable interest.

